# Economic evaluation of HIV pre-exposure prophylaxis among men-who-have-sex-with-men in England in 2016

**DOI:** 10.2807/1560-7917.ES.2017.22.42.17-00192

**Published:** 2017-10-19

**Authors:** Koh Jun Ong, Sarika Desai, Nigel Field, Monica Desai, Anthony Nardone, Albert Jan van Hoek, Owen Noel Gill

**Affiliations:** 1 HIV & STI Department, National Centre for Infectious Disease Surveillance and Control (CIDSC), Public Health England, London, United Kingdom; 2 Research Department of Infection & Population Health, University College London, London, United Kingdom; 3 London School of Hygiene & Tropical Medicine, London, United Kingdom

**Keywords:** human immunodeficiency virus - HIV, pre-exposure prophylaxis - PrEP, men who have sex with men – MSM, economic modelling, cost-effectiveness analysis

## Abstract

Clinical effectiveness of pre-exposure prophylaxis (PrEP) for preventing HIV acquisition in men who have sex with men (MSM) at high HIV risk is established. A static decision analytical model was constructed to inform policy prioritisation in England around cost-effectiveness and budgetary impact of a PrEP programme covering 5,000 MSM during an initial high-risk period. National genitourinary medicine clinic surveillance data informed key HIV risk assumptions. Pragmatic large-scale implementation scenarios were explored. At 86% effectiveness, PrEP given to 5,000 MSM at 3.3 per 100 person-years annual HIV incidence, assuming risk compensation (20% HIV incidence increase), averted 118 HIV infections over remaining lifetimes and was cost saving. Lower effectiveness (64%) gave an incremental cost-effectiveness ratio of + GBP 23,500 (EUR 32,000) per quality-adjusted life year (QALY) gained. Investment of GBP 26.9 million (EUR 36.6 million) in year-1 breaks even anywhere from year-23 (86% effectiveness) to year-33 (64% effectiveness). PrEP cost-effectiveness was highly sensitive to year-1 HIV incidence, PrEP adherence/effectiveness, and antiretroviral drug costs. There is much uncertainty around HIV incidence in those given PrEP and adherence/effectiveness, especially under programme scale-up. Substantially reduced PrEP drug costs are needed to give the necessary assurance of cost-effectiveness, and for an affordable public health programme of sufficient size.

## Introduction

In the United Kingdom (UK) new prevention initiatives are needed to reduce the estimated 2,800 incident HIV infections occurring annually in men who have sex with men (MSM) [1]. The UK PROUD study demonstrated that HIV pre-exposure prophylaxis (PrEP) with daily oral antiretroviral (ARV) drug combination tenofovir disoproxil and emtricitabine in addition to standard-of-care risk reduction for MSM at high HIV risk, reduced HIV incidence over the participant follow-up period by 86% (90% confidence interval (CI): 64–96%) [2]. The PROUD data on PrEP effectiveness, supported by the placebo-controlled efficacy data from iPrEX and IPERGAY, showed that PrEP offers a major opportunity to reduce HIV incidence in MSM [3,4]. A PrEP policy was proposed by National Health Service (NHS) England for high HIV risk attendees of the 215 genitourinary medicine (GUM) clinics in England that provide free, confidential, open-access sexual health services [5].

In England, new clinical commissioning policies are prioritised on their effectiveness and value for money [6]. Cost-effectiveness evidence is reviewed, with incremental value for money of competing services scored and compared on the basis of their incremental costs and incremental benefits. In other areas of publically funded public health prevention programmes (e.g. immunisation), one decision criterion used is a high certainty that the incremental cost-effectiveness ratio (ICER) falls below a recommended threshold, currently GBP 20,000 (EUR 27,210) per quality-adjusted life year (QALY) gained [7,8]. In addition, the affordability of any new service must be ensured based on practical eligibility criteria that are developed to guarantee the service reaches those with greatest need [6].

A static decision analytical model was used to explore the economic implications of a first phase scale-up of a PrEP programme for MSM GUM clinic attendees at high HIV risk, beginning in 2016. The method is valid for a modest scale initial PrEP programme with limited indirect (herd) effect [9], and was chosen for the relatively limited assumptions required, its transparency and ease of interpretation for decision makers, and because of the increasing uncertainties when estimating costs and effects after 5 to 10 years. Moreover, the technique was suitable because the impact on population disease dynamics is likely to be limited in the early years of a PrEP policy given the small numbers protected relative to the total at risk [9].

## Methods

The perspective of a healthcare provider was taken. A 5,000 person-years PrEP coverage level was judged to be reasonable for this initial scale-up period, based on the range suggested by a multidisciplinary, multi-stakeholder group of clinicians, patients, commissioners (budget holders) and public health practitioners [5]. The 4,500–6,500 range was generated after considering the evidence around likely programme roll-out scenarios, the GUM clinic activity dataset (GUMCAD) estimated need, patient-level uptake as informed by community surveys about willingness to take PrEP, and considered potential organisational challenges of delivery across many GUM clinics as well as evidence of PrEP scale-up in other countries [10].

**Figure 1:**
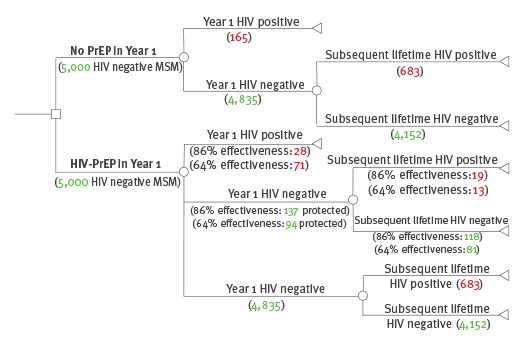
Decision analytical model structure comparing no-PrEP with PrEP in 5,000 men who have sex with men at high HIV risk^a^, England, 2016

MSM: men who have sex with men; PrEP: pre-exposure prophylaxis.

The lifetime HIV risk of 5,000 MSM who began an initial high HIV risk period of one year on PrEP was compared with the lifetime risk of the same group in the absence of PrEP (Figure 1). This required age distribution of MSM at high behavioural risk and estimates of HIV acquisition during the high-risk period of PrEP eligibility, as well as estimates of lifetime HIV acquisition, to account for the residual HIV risk after the high-risk period had passed. PrEP provision to a single high-risk year was modelled at the cohort-level. At the individual-level, should high risk continue beyond the first year, then that individual will form part of a new high-risk cohort in the second year. The ICER for PrEP remains the same for the second cohort as for the previous year’s high-risk cohort.

Red and green colours are used to indicate the estimated numbers of HIV-positive or negative MSM respectively, after a defined period (i.e. after the first year of PrEP (year-1) or, for the control group, after year-1 without PrEP, and for the remaining lifetime in both groups).

^a^ HIV incidence in Year-1 is 3.3 per 100 person-years and cumulative lifetime incidence without PrEP is 16.96%.

Data were extracted from GUMCAD [11], a comprehensive, pseudo-anonymised digital download of patient-level data on all sexually transmitted infection (STI) services and diagnoses provided in GUM clinics in England. Each pseudo-anonymised record contains a clinic identifier as well as a local patient number, so data from the same individual attending the same clinic can be linked longitudinally. Estimates of lifetime HIV risk were adjusted to the age-distribution of MSM GUM clinic attendees, using averages for years 2013–14 (see supplementary material [12])**.**

In the principal scenarios, MSM receiving PrEP were assumed to be prescribed daily tenofovir disoproxil and emtricitabine combined tablet, in accordance with the European Medicines Agency licensed prevention indication [13]. Event-based dosing (i.e. PrEP given before and after sexual exposure) for an average of four tablets used per 7-day period, was explored in sensitivity analyses [4].

Individuals given PrEP will be managed via GUM clinics; for a one year programme, each individual will have five visits to the clinic, at month 0, 1, 3, 6, and 9. The first visit includes assessment of clinical need for PrEP, confirmation of HIV and STI status, and measurement of renal function. Subsequent visits are for monitoring of drug adherence, tolerability, and safety, together with quarterly checking of HIV and STI status [2]. The additional elements of GUM clinic care directly attributable to PrEP were micro-costed (see supplementary material [12]).

### Estimating HIV incidence

GUMCAD data on HIV-negative clinic attending MSM for 2009 to 2013 were extracted. Diagnosis or not of any bacterial STI in the previous year was used to indicate recent condomless anal intercourse and to stratify the future risk of being diagnosed with HIV. Those with a bacterial STI in the previous year were labelled ‘high-risk’ and eligible for PrEP, and those without as having ‘medium-risk’ for HIV acquisition [14]. To estimate current HIV incidence in these strata, records were used of MSM with at least one additional documented HIV test between 43 to 365 days after the first HIV test documented in 2012, the most recent year with sufficient data (followed-up to end 2013) for analysis [14]. HIV incidence estimation methodology follows that used in Desai et al. [14]. 

MSM who did not attend a GUM clinic were assumed to be at ‘low-risk’ [14] (see also supplementary material [12]). To estimate HIV incidence in this stratum, total MSM numbers were calculated by combining the male proportion reporting same-sex partnerships in a 2010–12 national survey with 2012 male population estimates [15,16]. Estimated MSM living with HIV (diagnosed and undiagnosed) and GUM attending HIV-negative MSM were subtracted to get the denominator of those at low-risk [1]. Estimated HIV infections that occur in high- and medium-risk MSM were subtracted from the back-calculation estimate of all 2012 HIV infections in MSM to give the numerator for those at low-risk.

**Figure 2:**
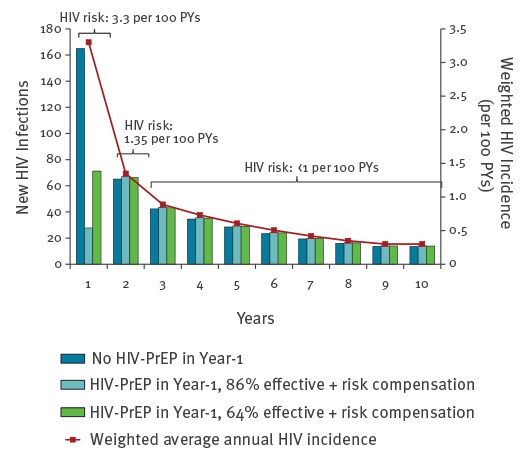
Impact of year-1 PrEP^a^ on HIV incidence over 10 years for 5,000 MSM at initial high HIV risk, England, 2016–2025

MSM eligible for PrEP begin at high-risk and move to medium- or low-risk at a changing probability. Lifetime HIV incidence combined movement between risk strata with estimated stratum-specific HIV incidence. Follow-up of high-risk MSM clinic attendees informed the proportions that stayed high-risk with bacterial STI diagnoses each year, those that became medium-risk who attended a clinic annually without bacterial STI diagnosis, and those without clinic attendance who became low-risk. Allowance was made for any transition from low- or medium-risk back to medium- or high-risk. If in 2013, x% of MSM who began as high-risk in 2009 remained high-risk, y% had become medium-risk, and z% low-risk, and HIV incidence was *Η*, *Μ*, and *Λ* for high, medium, and low-risk respectively, then the weighted average HIV incidence in 2013 was (x%*Η) + (y%*Μ) + (z%*Λ). Similarly calculated weighted HIV incidence averages were used for years 2010, 2011 and 2012. By assuming the same rate of change in risk from 2009 through 2013 and the same HIV incidence by risk stratum, future HIV incidence in 2017 through 2020 was estimated for MSM who began as high-risk in 2016 (PrEP programme year-1). After year-5 in 2020, future annual HIV incidence was interpolated using a constant rate of reduction until it reached *Λ*, and subsequently kept at *Λ* until age 75 years, after which risk of HIV acquisition was assumed to be zero. This approach created a declining HIV incidence over time. A slightly higher number remained susceptible in the PrEP group due to their PrEP protection during the first year. Therefore, over the subsequent lifetime to age 75 years, the absolute number of HIV infections each year was slightly greater in the PrEP group compared with the non-PrEP group (Figure 2).

PrEP: pre-exposure prophylaxis; PYs: person-years.

The bars on this chart represent the number of new HIV infections by year: (i) In the absence of PrEP (blue bars); (ii) If PrEP is given in year-1 and assuming 86% effectiveness + risk compensation (turquoise bars); (iii) and if PrEP is given in year-1 and assuming 64% effectiveness + risk compensation (green bars).

Up to age 75 years, there were 848 HIV infections without PrEP, and 730 with PrEP at 86% effectiveness and 767 with PrEP at 64% effectiveness. Slightly more later-HIV infections occur in those given PrEP during year-1 as the MSM protected by PrEP become susceptible on stopping PrEP, albeit at a much lower risk level.

^a^ PrEP effectiveness at either 86% or 64%, both with risk compensation adjustment (see text).

### Economic evaluation

A national guide for technology appraisals was followed [7]. PrEP users were assumed not to require HIV post-exposure prophylaxis following sexual exposure (PEPSE). Lifetime HIV infection care cost (excluding ARV costs) were stratified by CD4+ status at diagnosis [7,17]. HIV surveillance data were used to estimate average time to diagnosis once infected, CD4+ count at diagnosis, and rate of CD4+ recovery upon ARV commencement [18] (see also supplementary material [12]). Prompt initiation of ARV treatment following diagnosis was assumed [19].

Drug treatment costs used average 2013–15 NHS England ARV cost [20]. Future costs and QALYs were discounted annually by 3.5% and adjusted to 2014/15 GBP values (EUR values presented in parentheses, using year end 31 December 2015 historical exchange rates of GBP 1 equals EUR 1.3605) [7,21]. Economic parameters are presented in Table 1.

**Table 1:** Economic parameter estimates used in the two principal scenarios (providing PrEP or not), and value or range explored in sensitivity analyses, England, 2014/15 cost values

Parameter	Value	Sensitivity analyses range (min. to max. value of scenarios considered)	Explanatory notes and data source
Discount rate (cost)	3.5%	1.5% – 3.5%	[7]
Discount rate (QALYs)	3.5%	1.5% – 3.5%	[7]
**Costs**			
Annual cost of PrEP drug	GBP 4,331 (EUR 5,892)	GBP 433 – GBP 4,331 (EUR 589 – EUR 5,892) Discount range: 20% to 90%	[32] (last accessed 5 August 2016); price excludes VAT and was directly applied to the cost-effectiveness analysis
Annual cost of PrEP-related GUM tariffs	GBP 176 (EUR 239)	ND	[2,33], see also supplementary material [12]
PEPSE drug cost^a^ (averted in those taking PrEP)	GBP 772^a^ (EUR 1,050) per PEPSE course	NA	[32] (BNF last accessed 5 August 2016); price excludes VAT and was directly applied to the cost-effectiveness analysis
PEPSE GUM clinic costs (averted in those taking PrEP)	GBP 250 (EUR 340) per PEPSE course	NA	[33] (adapted to the current study)
Annual cost of an undiagnosed HIV infection	GBP 0 (EUR 0)	GBP 0 – GBP 2,499 (EUR 0 – EUR 3,400)	Assumption; GBP 2,499 based on HIV care costs for individuals diagnosed at CD4+ > 200 cells per mm^3^ not on ARV treatment^b^ [17,21]
Annual cost of ARV treatment per HIV-positive individual	GBP 4,741 (EUR 6,450)	Price reductions from 2019: range 0% to 80%	[20]^c^
Annual care cost of HIV + CD4 > 200 cells per mm^3^	GBP 4,734 (EUR 6,441)	ND	[17,21]
Annual care cost of HIV + CD4 < 200 cells per mm^3^	GBP 7,479 (EUR 10,175)	ND	[17,21]
Time to CD4+ recovery from < 200 cells per mm^3^	3 months	NA	Based on analysis of HIV data [18]
**QALY values**			
Disutility between HIV infection and diagnosis	0	0 – 0.11	Assumption [34]
Disutility associated with HIV infection – per annum	0.11	0.10 – 0.13	[34]
Utility values in UK men aged over 75 years^d^	0.75	NA	[35]

ARV: antiretroviral; BNF: British National Formulary; GUM: genitourinary medicine; max: maximum; min: minimum; NA: not applicable; ND: not done; NHS: National Health Service; NICE: The National Institute for Health and Care Excellence; PEPSE: post-exposure prophylaxis; PrEP: pre-exposure prophylaxis; QALY: quality-adjusted life year; VAT: value added tax; UK: United Kingdom.

^a^ This price represents the highest possible cost of current PEPSE drug recommended for use by NHS England (tenofovir disoproxil/emtricitabine/raltegravir) based on BNF list price, excluding VAT for the cost-effectiveness analysis in accordance with NICE Methods Guide.

^b^ Cost excludes specific HIV-related costs such as CD4+ and viral load measurements, and resistance testing (personal communication, V Cambiano, December 2015).

**^c^** Principal scenario used NHS England reported spend on ARV treatment. In sensitivity analyses, although actual timing of availability of generic ARVs for treatment is unknown, sensitivity analyses explored potential availability from 2019. This was based on the estimated patent expiration of individual compounds of the combination ARV treatment tenofovir disoproxil/emtricitabine/efavirenz (proprietary name: Atripla) by 2018 [36]. Combination tenofovir disoproxil/emtricitabine/efavirenz is one of the British HIV Association preferred choice of ARV treatment to begin with in therapy-naïve patients [37].

**^d^** We assumed that an HIV-positive individual has a life-expectancy of 75 years [38]. Given that the life-expectancy at birth for males in England (2010 to 2012 Office for National Statistics estimates) was 79 years, this meant that an HIV-positive individual who dies at age 75 years would have lost four years of quality of life [16]. We combined this last four years with the utility values among UK men aged above 75 years (0.75 per year), which was obtained from the EQ-5D utility values for UK male population, to obtain the QALY losses during these final four years of life lost consequent to earlier deaths related to HIV [35].

Model outputs included number of new HIV infections and the ICER, as cost per QALY gained, of PrEP compared with no PrEP. Budget impact analyses were presented in present 2014/15 values and included value added tax (VAT: + 20%) on PrEP drug costs [7]. PrEP service investment time to break-even was calculated as the years to when the cumulative savings from HIV infections averted in year-1 began to exceed PrEP costs in year-1.

### Risk compensation

Published evidence suggests increased frequency of condomless anal sex subsequent to PrEP use and increased STI diagnoses [2,22]. Risk compensation would also lead to an increase in HIV exposure. With PrEP scale-up, adherence may reduce and thereby increase HIV transmission. To explore risk compensation, an arbitrary increase of HIV incidence by 20% in those given PrEP was assumed in the principal scenarios. At 64% PrEP effectiveness, for example, annual HIV incidence is (100% – 64%) * *Η* = 36% * *Η*, where, *Η* = HIV incidence in high-risk MSM. If *Η* is increased by 20% due to risk compensation, then annual HIV incidence becomes (100 – 64%)*(100% + 20%) *Η = 43.2% * *Η*.

### Sensitivity analyses

Sensitivity analyses explored plausible ranges of key parameter values (Table 1). Univariate sensitivity analyses were based on cautious choices considered more plausible with substantial scale-up. The scenario with 64% PrEP effectiveness and risk compensation was the preferred benchmark and corresponding ICERs were plotted on a tornado diagram.

**Figure 3:**
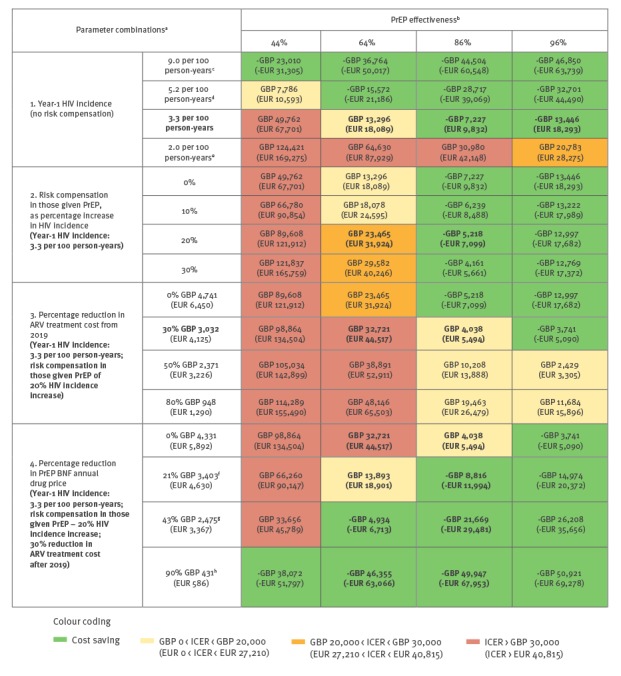
Multivariate sensitivity of incremental cost-effectiveness ratio (ICER) for different levels of pre-exposure prophylaxis (PrEP) effectiveness, England, 2014/15 cost values

BNF: British National Formulary; GUM: genitourinary medicine; GUMCAD: GUM clinic activity dataset; MSM: men who have sex with men; STI: sexually transmitted infection; UK: United Kingdom.

Multivariate sensitivity analyses were conducted to illustrate the margin of certainty around whether or not PrEP would remain cost-effective, at different PrEP effectiveness level (Figure 3). Due to the nature of the uncertainties, full probabilistic sensitivity analysis was not possible.

**^a^** First parameter combination (i.e. Year-1 HIV incidence of 3.3 per 100 person-years) assumed within second combination, first and second within third, etc.

**^b^** 44% was the efficacy level reported in the iPrEx trial; 86% was the UK PROUD trial observed clinical effectiveness level, while 64% and 96% were the lower- and upper-bound 90% confidence intervals reported in this latter trial [2,3].

**^c^** Reported HIV incidence in the deferred part (no PrEP, n = 267 MSM) of the PROUD trial [2].

**^d^** Estimated HIV incidence in HIV-negative MSM with documented rectal bacterial STI diagnosis in 2012, GUMCAD analysis.

^e^ Estimated HIV incidence in all HIV-negative MSM GUM attendees in 2012, GUMCAD analysis.

**^f^** 21% reduction in PrEP drug price due to 50% event-based dosing i.e. prorated 5.5 tablets per 7-day. This assumed that if an MSM was prescribed event-based dosing, then only four tablets would be dispensed for every 7-day i.e. 4/7 of the drug cost. Event-based dosing frequency based on the findings reported in the IPERGAY trial [4]. Service provision through GUM clinics remained the same, as frequency of monitoring remained the same.

**^g^** 43% reduction in PrEP drug price due to 100% event-based dosing (four tablets per 7-day).

**^h^** 90% reduction in PrEP drug price due to fall in current tenofovir disoproxil/emtricitabine price following patent expiry of the PrEP drug used. This is an arbitrary assumption. The future price is dependent on market competition. The exact timing of when this will happen, however, is uncertain. The patents for tenofovir disoproxil and emtricitabine expired in 2016 and July 2017, respectively. However, Truvada as a combination tablet containing both tenofovir disoproxil/emtricitabine has a supplementary protection certificate (SPC) providing market exclusivity protection until February 2020, although this SPC is being challenged [39].

## Results

An estimated 466,000 HIV-negative MSM aged between 15 to 75 years-old live in England in 2012, 85,500 (23%) of whom attended GUM clinics during that year. A fifth of the 85,500 (17,400 MSM) had a documented bacterial STI diagnosis i.e. proxy for high risk. Over time, GUMCAD data have shown an increase in the number of HIV-negative MSM GUM attendees, as well as the subset diagnosed with bacterial STI. Thus, the 5,000 person-years PrEP covered 29% of the GUMCAD identified high-risk cohort (who may not represent all high-risk MSM as not all attended GUM clinics [23]), 6% of all HIV-negative MSM GUM attendees, and just 1% of the estimated HIV-negative MSM population in England.

The HIV incidence observed in the high-risk PrEP-eligible stratum was 3.3 per 100 person-years (95% CI: 2.8–4.9 per 100 person-years), and 1.5 per 100 person-years (95% CI: 1.3–1.8 per 100 person-years) in the medium-risk stratum. In the low-risk MSM stratum the indirectly estimated HIV incidence was 0.3 per 100 person-years (Table 2). The HIV incidence estimates showed that GUM attending MSM had higher HIV risk than non-GUM attending MSM.

**Table 2:** Population size and HIV incidence in men having sex with men (MSM), England, 2012

HIV-negative MSM by risk stratum	MSM numbers^a^	Annual HIV incidence, per 100 person-years (95% CI)	Annual HIV infections^a^
**a. HIV incidence in GUM clinic attendees (directly estimated^b^)**			
High-risk – GUM clinic attendees with bacterial STI in previous year and/or at first attendance of year	17,400	3.3 (2.8*–*4.9)**^c^**	570^d^
Medium-risk – GUM clinic attendees with no recorded bacterial STI in previous year or at first attendance of year	68,100	1.5 (1.3*–*1.8)**^c^**	1,020^d^
**b. Overall HIV incidence, England**			
PHE back-calculation^e^			2,790
**c. HIV incidence in non-GUM clinic attendees (indirectly estimated^f^)**			
Low-risk – HIV-negative non-GUM clinic attendees	395,000	0.3	1,200

CI: confidence interval; GUM: genitourinary medicine; ONS: Office for National Statistics; MPES: multi-parameter evidence synthesis; Natsal: National Survey on Sexual Attitudes and Lifestyles; PHE: Public Health England; STI: sexually transmitted infection.

^a^ Numbers rounded to three significant figures or nearest 10.

**^b^** Estimated using 2013 Genitourinary Medicine Clinical Activity Dataset – GUMCAD [11].

**^c^** As observed in re-attending sub-group.

**^d^** Applying observed incidence to whole group and rounded to three significant figures or nearest 10.

^e^ Year 2012 estimated numbers, using methodology as described in Birrell et al., 2013 [30].

**^f^** Estimated 1,200 annual infections calculated by deducting number of infections among GUM clinic attendees (high-risk and medium-risk; 1,595) from overall annual HIV incidence (2,790) [30]. Low-risk population size of 395,000 (i.e. non-GUM attending MSM) estimated using a combination of MPES (England and Wales, aged 15–44 years), ONS (England and Wales population estimates for mid-2012), and Natsal-3 (proportion of MSM by age group), to obtain an estimate of the non-GUM attending MSM population in England for ages 15–74 years [1,15,16]. HIV incidence in this low-risk group (1,200 annual infections per 395,000 population) rounded to 0.3 per 100 person-years.

Of the 11,742 MSM without diagnosed HIV and with a recent bacterial STI (proxy for high HIV risk), who attended clinic in 2009 (the first of a five year, 2009–2013, longitudinal analysis), only 26% were categorised as high-risk in 2010. This decrease in the proportion of the initial 2009 attendees categorised as high-risk in subsequent years continued through 2011, 2012 and 2013, to 10%, 7% and 5% (see supplementary material [12]). Consequently, there was a large reduction in the weighted average annual HIV incidence for year-2 to year-5 (Figure 2). Interpolating the declining risk behaviour in the cohort and subsequent HIV acquisition forward, the annual HIV incidence reached the lower risk tier of 0.3 per 100 person-years annually by year-9, after which it was kept constant until age 75 years.

Combining the weighted average annual HIV incidence for MSM and the age distribution of MSM clinic attendees in 2013 and 2014, the estimated lifetime HIV incidence to age 75 years in an MSM clinic attendee who began year-1 at high-risk, was 16.96%.

Applying a 20% HIV incidence increase to those given PrEP in year-1, as a risk compensation adjustment, the estimated cumulative HIV incidence to age 75 years was reduced from 16.96% (no PrEP) to 15.4% at 64% PrEP effectiveness, while at 86% effectiveness, it fell to 14.6%.

After the year-1 high-risk period, HIV incidence reduced to 1.35 per 100 person-years and PrEP was no longer indicated. Moreover, a small fraction of those who were protected by PrEP during the first year became infected later in life. The contribution of PrEP, given only during the year-1 high-risk period, to reducing lifetime HIV risk was modest, impacting on close to 20% of lifetime risk, because of the relatively short period that MSM remained at high risk (see supplementary material [12]).

### Economic evaluation

Without PrEP in year-1, an estimated 848 HIV infections occurred, producing future discounted HIV care costs of GBP 84.3 million (EUR 115 million), and a loss of 1,830 QALYs (discounted). Almost half of these infections occurred within the first 10 years (see supplementary material [12]).

Assuming daily PrEP at 86% effectiveness (with risk compensation), an estimated 730 lifetime HIV infections occurred. Year-1 PrEP cost (drug and GUM clinic) of GBP 22.5 million (EUR 30.7 million) prevented GBP 24.1 million (EUR 32.9 million) HIV care costs (discounted) and GBP 256,000 (EUR 348,000) PEPSE-related costs, saved 361 QALYs (discounted), and over a lifetime was cost-saving (i.e. ICER is negative), compared with no PrEP. Delivering PrEP to 5,000 high-risk MSM resulted in 137 less year-1 HIV infections. However, 19 of these 137 acquired HIV while at medium- or low-risk later in life, reducing the total infections prevented to 118. Nevertheless, these 19 infections were delayed with corresponding reductions in costs and QALY losses.

At 64% PrEP effectiveness (with risk compensation), the lifetime HIV infections were 767. Year-1 PrEP service cost of GBP 22.5 million (EUR 30.7 million) prevented GBP 16.5 million (EUR 22.4 million) HIV care costs (discounted) and GBP 256,000 (EUR 348,000) PEPSE-related costs, and saved 247 QALYs (discounted). Under this scenario, the ICER increased to + GBP 23,500 (EUR 31,900), just above the current cost-effectiveness threshold for England [7]. The reduced effectiveness gave 94 less year-1 HIV infections, although 13 of the 94 acquired HIV in later years.

**Figure 4:**
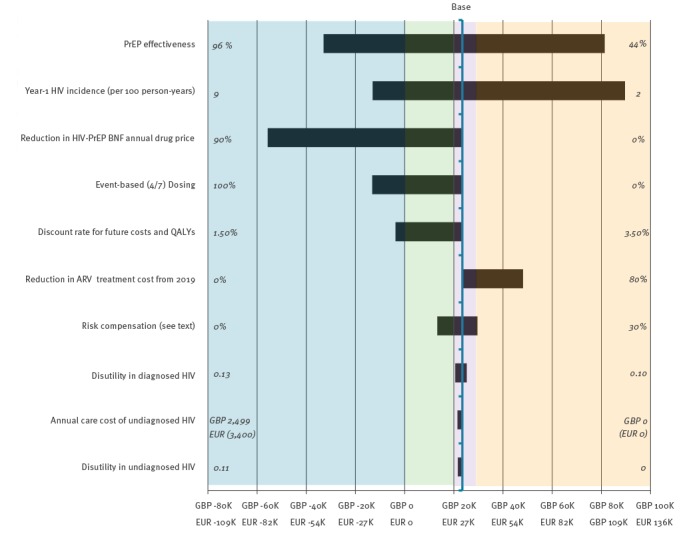
Univariate sensitivity of pre-exposure prophylaxis (PrEP) incremental cost-effectiveness ratio (ICER) around base case^a^ for plausible ranges^b^ of key parameters, 2014/15 cost values

ARV: antiretroviral; BNF: British National Formulary; QALY: quality-adjusted life year.

^a^ Base case ICER + GBP 23,500 (EUR 31,900) per QALY gained, set at 64% PrEP effectiveness and a 20% increase in HIV incidence in those given PrEP due to risk compensation (see text).

The ICER was very sensitive to assumptions about HIV incidence in the PrEP eligible group, PrEP effectiveness when scaled-up, PrEP drug costs, and future reductions in the cost of ARV treatment (Figure 4).

**^b^** Extremes of parameter ranges shown at either end of horizontal bars.

PrEP was much less cost-effective if HIV incidence was 2 per 100 person-years (the estimated overall HIV incidence in MSM GUM clinic attendees), or if PrEP effectiveness dropped to 44%. Similarly, albeit to a lesser extent, reduced future treatment costs produced a less favourable ICER for PrEP. However, a more favourable ICER resulted through reducing PrEP drug costs, either through price reduction or reduced dosing frequency from daily to event-based.

If, under scale-up, PrEP stayed 86% effective with 20% HIV risk increase (risk compensation adjustment), then for most parameter combinations a PrEP policy stays cost-effective, unless the eligible group HIV incidence was 2 per 100 person-years or less (Figure 3). If, however, effectiveness was 64% with the same degree of risk compensation, then a PrEP service will only be cost-effective if year-1 incidence is over 3.3 per 100 person-years and there is no change in future HIV treatment costs (i.e. ignoring availability for treatment of generic ARVs by 2019, see notes for Table 1), or if PrEP drug cost is reduced.

### Budgetary implications

In a single year, a PrEP service of 5,000 person years cost GBP 26.9 million (EUR 36.6 million), at current British National Formulary (BNF) list price (inclusive of 20% VAT). As HIV care costs accrued over time, it took many years before investment in the first year was recovered. At 86% PrEP effectiveness, it took 23 years of cumulative savings from HIV care cost averted for the year-1 investment to break even and 33 years if PrEP was only 64% effective, both assuming risk compensation.

If there was a substantial reduction in PrEP drug price (e.g. by 90%), the budget to cover 5,000 person years became GBP 3.48 million (EUR 4.73 million). Break-even of year-1 investment happened by the fifth year at 86% effectiveness or by the sixth year at 64% effectiveness, again assuming risk compensation.

Time to break-even of the initial year of PrEP was extended should future HIV treatment costs reduce. At current BNF list price, a 30% reduction in future ARV treatment costs from 2019 onwards increased the time to break-even of the one-year investment in PrEP in 2016 to 38 years, assuming 64% PrEP effectiveness with risk compensation.

## Discussion

Oral PrEP given to MSM at high HIV risk, assuming good adherence and correspondingly high clinical effectiveness, was potentially cost-effective in England. The ICERs, however, were very sensitive to key parameters such as the risk of HIV for PrEP recipients and adherence (effectiveness). When PrEP is scaled-up to service provision level there is doubt that the values for these parameters observed in clinical trial settings will apply. Moreover, at the current BNF price the budgetary impact of a modest annual programme of 5,000 PrEP person years was considerable.

The cost-effectiveness of PrEP scale-up depends first on reaching those at high risk of HIV, who need to be identified, offered and to accept PrEP; if many at medium-risk take PrEP, HIV incidence in those taking PrEP will be overestimated. Second, PrEP adherence may be lower with scale-up than in smaller clinical trials; results from ‘real-world’ effectiveness trials, which generally recruit committed early adopters who may be at exceptionally high-risk, may not be repeated in programmes for all at high-risk [24]. Third, there is uncertainty about whether or not condomless anal intercourse frequency will increase in those given PrEP (risk compensation), leading to more exposures and increased HIV in those with poor PrEP adherence as well as increased bacterial STIs and hepatitis C, thus blunting PrEP benefit; so far a possible HIV incidence increase mediated through diminished adherence during scale-up has not been observed, but there is emerging evidence suggesting risk compensation and bacterial STI increase in those on PrEP [25]. Sensitivity analyses of plausible combinations of these factors did not give a high degree of certainty that the ICER for PrEP would be below GBP 20,000 (EUR 27,210) per QALY gained [7]. Moreover, despite differences in model structure and input assumptions that were appropriate for England, these findings were broadly in agreement with economic evaluations from other high income countries [26-29], which found ICERs to be highly dependent on HIV incidence, costs of the PrEP drug and adherence-related effectiveness. This analysis highlights critical considerations for PrEP implementation in other European countries, even if their HIV epidemic is different, as the problems arising around implementation, financial considerations, and programme sustainability are common.

A key strength of this study was the use of empirical data on many thousands of MSM attending GUM clinics in England over a contemporary period to measure HIV incidence and risk turnover. A critical assumption was that future HIV incidence in MSM GUM clinic attendees will replicate that observed between 2009 and 2013, and further consequences of any recent changes in sexual behaviour were not captured. However, it was reasonable to extrapolate forward current HIV incidence estimates given the scale and timeliness of the source GUMCAD data and the recent back-calculation estimates that showed no change in overall HIV incidence in MSM [1,30].

A major limitation of our analysis was the use of a static decision analysis approach instead of a dynamic transmission model, as it did not quantify the benefit of PrEP on the wider HIV epidemic in England, including the benefits for those not given PrEP. Therefore, there was an underestimation of the total benefit. Nevertheless, since our cohort of 5,000 MSM was very small (1%) compared with the overall HIV-negative MSM population in England, and modest compared with the higher risk group of GUM attendees (29%), the likely indirect impact of the PrEP programme would be limited. Recently, Nichols et al. quantified this indirect effect of a similarly modest PrEP intervention (average 4,500 MSM annually) delivered to a Dutch MSM population using a dynamic model and showed only a 13–16% change in the ICER when indirect effects were included [26]. Therefore, with a modest programme, the majority of benefits fall on those given PrEP. Should a very large PrEP programme be implemented, the long-term indirect effects would increase in dominance and a static modelling approach would be inappropriate.

In conclusion, whether or not PrEP drug is priced at a level that guarantees favourable cost effectiveness, reduced budgetary impact, and a shorter return on investment period, the analysis highlights other questions about PrEP scale-up that directly affect the financial considerations and the sustainability of any future programme. When proposed high-risk eligibility criteria are implemented, who and how many will access and take up PrEP? Will PrEP be taken up by those in whom PrEP is clinically recommended? What will be their level of adherence? What will be the effectiveness of regular clinical risk assessment at assuring that only those at continuing high-risk stay on PrEP to maintain cost-effectiveness and equitable access based on clinical need? These questions should be answered before embarking on a long-term PrEP-based intervention. A further clinical trial is proposed as a means to do this [31].
